# Supplementary material for: Latent sub-structural resilience mechanisms in temporal human mobility networks during urban flooding
Source: Sci Rep. 2023 Jul 6;13:10953. doi: 10.1038/s41598-023-37965-6 (PMC10326012; doi:10.1038/s41598-023-37965-6)
Supplement: Supplementary file 1 — Supplementary Information. [file 41598_2023_37965_MOESM1_ESM.pdf]

Appendix

The following figure shows the motif conversion patterns for different days of the week. We observe that these conversion patterns are similar for same days of the week. Due to this similarity in motif conversion patterns for different days of the week, in this paper, we plot conversion trends for all motif types including symbolic motif type 0 for different days of the week, separately. Figure A1 also shows the changes in specific days before impact, during Harvey and immediate recovery. Days of impact show distint conversion trends for all motifs.

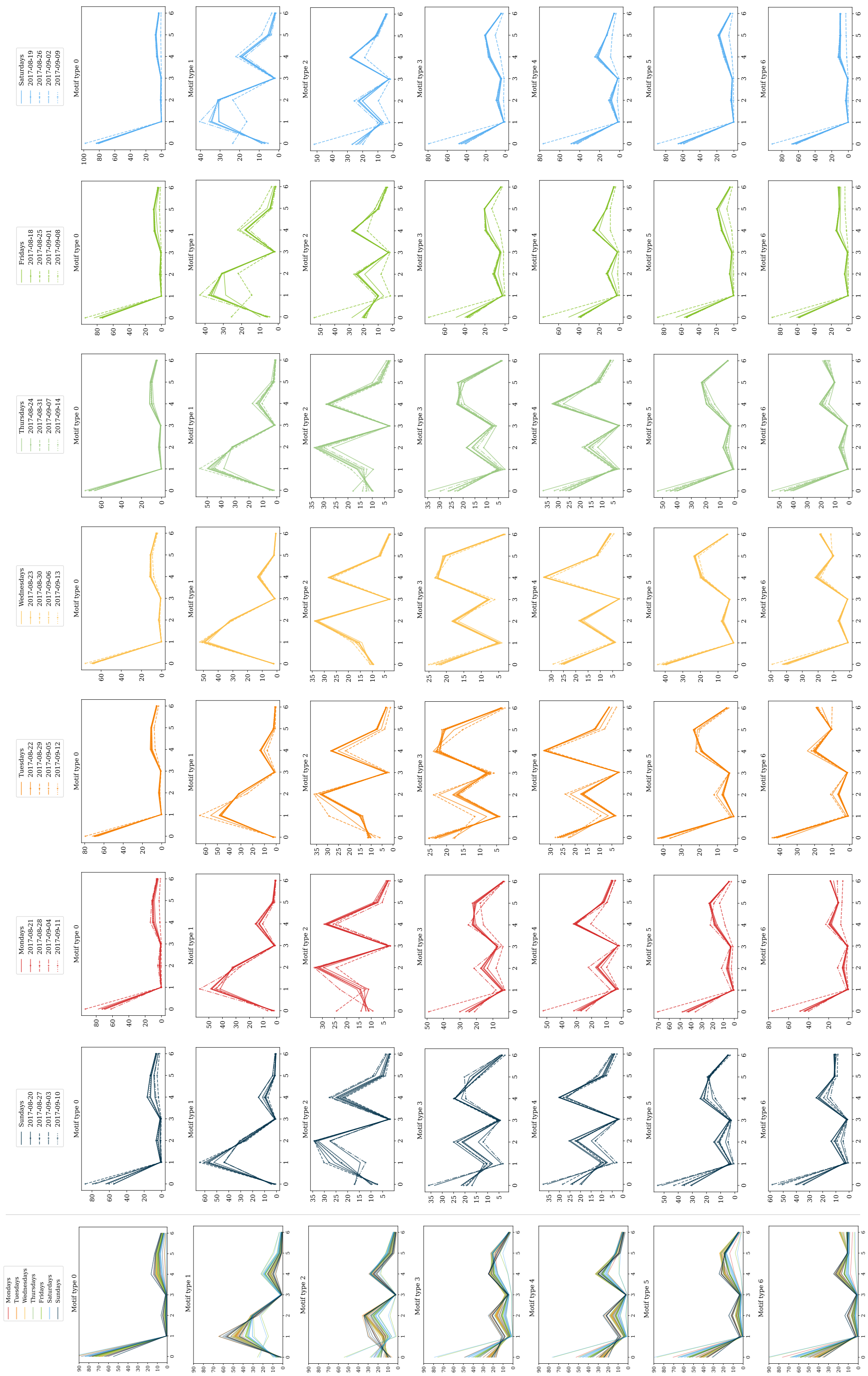

Figure A1. Motif conversion trends for different days of the week: first column shows the plots of all the days, with each day of the week color coded separately. Other columns show motif conversions for different days of the week and some days before, during, and immediately after the impact are highlighted for better interpretation of the results.
